# The impact of Chinese investment on access to clean and sustainable energy in Africa

**DOI:** 10.1016/j.heliyon.2024.e33991

**Published:** 2024-07-02

**Authors:** Dejun Zhou, Noha Alessa, Vincent Konadu Tawiah

**Affiliations:** a School of Law, Jiangsu University, Zhenijang, 212013, Jiangsu, China; b Department of Accounting, College of Business Adminstration, Princess Nourah Bint Abdulrahman University, P.O. Box 84428, Riyadh, 11671, Saudi Arabia; c Dublin City University, Ireland

**Keywords:** Africa, China, Sustainable electricity natural resources, Sustainable development

## Abstract

The objective of this study is to examine the relationship between Chinese investment and access to clean and sustainable energy in African countries. We are motivated by the increasing engagement between China and Africa and the priority of sustainable energy for both partners. We employ robust econometric modelling, including fixed effects and the two-step Generalized Method of Moments, on a panel sample of 43 African countries over 19 years. The results show that Chinese energy investment in Africa is significantly associated with increased access to sustainable electricity. This effect is more pronounced in resource-rich countries, suggesting that China is more attracted to these countries due to the opportunities for resource trade in the form of partnerships. The policy implication of this finding is that it highlights the importance of African governments developing favourable strategies and policies to attract more Chinese investments in the energy sector.

## 1 Introduction

In recent years, China has emerged as a major development partner for Africa, providing various forms of financial support across different sectors [1,2]. A significant portion of this support has been directed towards the energy sector. As a global leader in sustainable energy development and production, China is well-positioned to offer such services to its development partners [3,4]. China possesses the resources and expertise to develop various sustainable energy solutions, such as solar panels and hydropower [5,6]. Furthermore, compared to their Western counterparts, Chinese firms are technologically advanced and cost-effective in infrastructure projects [7]. Unlike investments from the USA and Europe, China's engagement in Africa has often centred around natural resources, leading to projects predominantly located on the outskirts of cities. This focus increases the likelihood of enhancing energy access in these areas to support operations and foster positive relations with local communities.

The Atlantic Council estimates that over the last decade, China's investment in energy infrastructure in Africa has increased tenfold [8]. Notable examples include the Buai Dam in Ghana, the Garissa photovoltaic power generation project in Kenya, the Aysha wind power project in Ethiopia, and the Kafue Gorge hydroelectric station in Zambia [9]. These projects highlight China's significant investment in Africa's energy sector. However, these investments may not always lead to increased access to clean and sustainable electricity in both urban and rural areas of the host countries for several reasons. First, China is known for exporting inferior goods and gadgets to developing countries [10]. Previous studies argue that a key difference between Western and Chinese investments is the quality and durability of the projects. Some suggest that China prioritises quantity over quality, potentially resulting in investments that do not last long enough to achieve the intended outcomes. Second, Africa's pervasive corruption and ineffective governance could hinder the expected benefits of these investments [11]. Unlike Western countries, China is less concerned with governance and corruption in host countries. For example, Martins et al. (2023) report a negative association between Chinese outward FDI and business freedom.

Given these unique characteristics of the China-Africa relationship and the significant Chinese investment in Africa's energy sector, it is important to explore whether this investment significantly impacts the continent. Therefore, this paper examines whether Chinese investment influences access to clean and sustainable electricity in Africa. We employ robust econometric identification strategies on a large sample of 43 African countries between 2003 and 2019. Our findings reveal a positive and significant association between Chinese investment and access to clean and sustainable energy in Africa, suggesting that Chinese investment increases the production and accessibility of clean and sustainable electricity in most African countries.

These findings contribute to the literature and offer policy implications. First, we extend the literature on the China-Africa relationship [[12], [13], [14], [15]] by examining the critical area of clean and sustainable energy. Over the years, China has positioned itself as a solution to Africa's energy crisis and a global leader in sustainable energy [16]. However, there is little empirical evidence on how Chinese investment drives changes in Africa's energy sectors. Our study provides new empirical evidence on the relationship between China's presence in Africa and clean energy production. We also contribute to the literature on foreign direct investment in sustainable energy production in developing countries. Unlike prior studies [17,18], we provide comprehensive findings by examining rural and urban areas, as well as resource-rich and non-resource-rich countries. This cross-sectional analysis demonstrates that some areas are more likely to benefit from foreign direct investment, particularly Chinese investment, in terms of access to clean energy in Africa.

Regarding policy implications, our study highlights that Chinese investment in Africa presents both opportunities and challenges. While these investments increase access to clean energy, contributing to the attainment of Sustainable Development Goals (SDG 7) and the Sustainable Energy for All Initiative, it is crucial for African countries to consider the terms of investment agreements carefully. Ensuring these agreements align with long-term development goals and promote sustainable economic growth is essential.

The remainder of the paper is structured as follows. In section 2, we provide context and arguments on how Chinese investment may influence access to clean and sustainable energies. Section 3 contains the research methodology, which includes econometric modelling. The results and discussions are presented in Section 4. Section 5 concludes the paper with policy implications and suggestions for future research.

### 1.1 Literature review

Access to all forms of energy, particularly electricity, is low in Africa [17,18]. It is estimated that less than half of the population in most African countries has access to electricity, a figure significantly lower compared to other developing regions [3,17,18]. Despite these low figures, the past decade has seen a rise in access to clean and sustainable electricity in Africa. For instance, the IEA reports that the yearly increase in electricity access in Africa doubled from 9 million people in the early 2000s to 20 million people between 2014 and 2018 (IEA, 2019; Mbiankeu Nguea et al., 2022). During this same period, Chinese investment in Africa has also increased. Data from a sample of 43 African countries indicates that China invested about 469.3 million USD annually between 2003 and 2021. China has indeed surpassed the USA and Europe as Africa's leading investment partner [2,8,19].

Over the last decade, compared to other forms of foreign direct investment, China has engaged in relatively large projects in the energy sectors of many African countries, significantly influencing access to clean and sustainable energy on the continent [9,20,21]. For example, the Buai Dam in Ghana and the Kafue Gorge hydroelectric station in Zambia provide clean electricity from water. The Garissa photovoltaic project in Kenya and the Sakai Photovoltaic project in the Central African Republic generate power from the sun. The Aysha Wind Farm Project in Ethiopia is another notable Chinese clean energy project on the continent.

Although other sources of foreign investment have also impacted access to and production of energy in Africa [22], Chinese investments bear some unique characteristics that are likely to drive a more substantial impact. First, Chinese investments in renewable energy projects often introduce technologies and equipment at competitive prices [23,24]. This can reduce the cost of energy production, making sustainable energy more affordable and accessible to a larger portion of the population, and improve renewable energy production while reducing carbon emissions [25].

Second, Chinese investments often include technology transfer and knowledge-sharing components. This can help build local capabilities in sustainable energy development, operation, and maintenance. Some Chinese investments feature capacity-building components, such as training local technicians and engineers, which enhance the skills needed to operate and maintain energy infrastructure [26]. China is known for exporting not only capital but also labour [1,27]. Unlike Western counterparts, the Chinese invest considerable time and labour in their projects, with many staying long-term to ensure a smooth transfer of technology to local personnel, even years after project completion [28,29].

Third, through its vision and mission of delivering climate commitments by increasing energy transition from fossil fuels, China has positioned itself as the global leader in sustainable energy production, specialising in hydro and solar power projects (Voice of America, 2023). China has been involved in funding and building large-scale hydropower projects in some African countries (China Daily, 2022; Mukherjee & Plessis, 2023). Additionally, China promotes solar energy in Africa with initiatives like the Solar Power Plan for Africa, aiming to provide off-grid and decentralised solar solutions to remote and underserved areas. China's manufacturing and supply chain capacities for these sustainable energy productions, such as producing solar panels and other renewable energy equipment, have contributed to cost reductions, making these technologies more accessible to African countries.

Fourth, unlike investments from the USA and Europe, which are largely privately funded, Chinese investments are often backed by state-owned banks and institutions. This support can facilitate the development of sustainable energy projects that might struggle to secure funding through traditional channels (Alden, 2015; Shen, 2015). Due to government backing, Chinese investors might be more willing to undertake risky projects with long gestation periods. Additionally, China's foreign policy of non-interference in the governance of host countries aligns with the interests of many African nations, fostering a sense of collaboration and facilitating project implementation.

## 2 Methodology

### 2.1 Data and sample selection

Given that the focus of this study is on Africa, we begin the sample selection with all 55 recognized African Union countries. The sample selection is based on data availability. We exclude countries without data on Chinese investment, such as Eritrea and South Sudan. Additionally, we exclude countries with insufficient data on the key independent variable (clean fuel and technology). After this careful elimination process, our final selection includes 43 countries over a 19-year period (2003–2021). The sample period is constrained by the availability of data on Chinese investment, which begins in 2003. The data were collected from reputable sources, including the China-Africa Research Initiative, the Sustainable Energy for All Initiative at the World Bank, and World Development Indicators. Table 1 lists the sample countries.

**Table 1:** List of countries.

Resource-rich countries	Non-resource rich countries	
Algeria	Angola	Niger
Cameroon	Benin	Rwanda
Chad	Burkina Faso	Senegal
Congo, Dem. Rep.	Burundi	Seychelles
Cote d'Ivoire	Central African Republic	South Africa
Equatorial Guinea	Egypt, Arab Rep.	Togo
Botswana	Eswatini	Tunisia
Gabon	Ethiopia	Uganda
Ghana	Gambia, The	Zimbabwe
Nigeria	Guinea	
Congo, Rep.	Guinea-Bissau	
Mali	Kenya	
Mauritania	Lesotho	
Mozambique	Madagascar	
Sierra Leone	Malawi	
Tanzania	Mauritius	
Zambia	Morocco	

### 2.2 Definition of variables

**China investment**: Over the last decades, China has used different routes to invest in Africa, including, construction contracts, trade, foreign aid and direct investment. While all these routes may be connected to the Chinese influence in Africa, foreign direct investment remains the significant form of investment which gives China more control over the outcome of its engagement [11]. This is because foreign direct investment offers direct control over projects and long-term relationships. Therefore, we measure China's investment as the amount of foreign direct investment stock in a sample country. For easy comparison among the sample countries, we mitigate the effects of size using the natural logarithm form. Consistent with prior studies [15,19] We collected data on Chinese investment from the China Africa Research Initiative (2020) at Johns Hopkins University.^1^

**Access to electricity**: We measure access to sustainable energy utilities as the number of people with electricity, which is the proportion of the total population of the country. Contrarily to prior studies, such as [17] who focused on only access by the total population, we capture access across both urban and rural areas. Therefore, we use three alternative measurements of access to sustainable energy utilities. First is the percentage of the total population with access to sustainable energy, which includes all people regardless of their location. Second, we use the same measurement but limited to people in the urban areas. The third measurement focuses on the percentage of rural population with access to sustainable energy utilities. All three variables are measured in percentage and are sourced from the Sustainable Energy for All Initiative hosted by the World Bank.

**Control variables**: In line with prior studies [17,18,30], we include batteries of control variables to account for factors that may drive access to sustainable energy utilities in Africa. Consistent with prior studies [31], we include gross domestic product (GDP) to control the effect of economic size while we include GDP per capita and GDP growth to control for economic development and economic growth, respectively. Arguably, countries with large economies and high development are more likely to have high access to sustainable energy because they have the resources to invest in those areas. We also include domestic credit and gross domestic savings to control the availability of financial resources in the country. Consistent with the assumption that international trade facilitates the transfer of energy technology into developing countries [30], we include trade openness to control the impact of international trade on access to sustainable energies [32]. The description and source of variables are presented in Table 2.

**Table 2:** Variable description and sources.

Name	Definition	Source
Chinese investment	The amount of foreign direct investment stock in a sample country. For easy comparison among the sample countries, we mitigate the effects of size using the natural logarithm form	China-Africa Research Initiative
Clean and sustainable energy	For cross sectional analysis purpose, we use three alternative measurement. Total: Percentage of total population with access to clean electricity. Urban: Percentage of urban population with access to clean electricity Rural: Percentage of rural population with access to clean electricity.	Sustainable Energy for All
Gross domestic product	GDP at purchaser's prices is the sum of gross value added by all resident producers in the economy plus any product taxes and minus any subsidies not included in the value of the products. It is calculated without making deductions for depreciation of fabricated assets or for depletion and degradation of natural resources. Data are in current U.S. dollars. Dollar figures for GDP are converted from domestic currencies using single year official exchange rates. For a few countries where the official exchange rate does not reflect the rate effectively applied to actual foreign exchange transactions, an alternative conversion factor is used.	World Development Indicators
GDP per capita	GDP per capita is gross domestic product divided by midyear population. GDP is the sum of gross value added by all resident producers in the economy plus any product taxes and minus any subsidies not included in the value of the products. It is calculated without making deductions for depreciation of fabricated assets or for depletion and degradation of natural resources. Data are in current U.S. dollars.	World Development Indicators
GDP growth	Annual percentage growth rate of GDP at market prices based on constant local currency. Aggregates are based on constant 2015 prices, expressed in U.S. dollars. GDP is the sum of gross value added by all resident producers in the economy plus any product taxes and minus any subsidies not included in the value of the products. It is calculated without making deductions for depreciation of fabricated assets or for depletion and degradation of natural resources.	World Development Indicators
Gross domestic savings	Gross domestic savings are calculated as GDP less final consumption expenditure (total consumption).	World Development Indicators
Domestic credit	Domestic credit to private sector refers to financial resources provided to the private sector by financial corporations, such as through loans, purchases of nonequity securities, and trade credits and other accounts receivable, that establish a claim for repayment.	World Development Indicators
Trade openness	The sum of export and import as a percentage of gross domestic product	World Development Indicators

### 2.3 Pre-regression and econometric modelling

To ensure the robustness of the results and mitigate the potential for biased results, we perform several pre-regression checks to test the reliability and validity of the dataset. We begin with a correlation matrix to check for potential multi-collinearity issues. The results of the Pearson pairwise correlation matrix are presented in Table 3. We observe a high correlation between the three measurements of sustainable energy. The correlation confirms the suitability of using these measurements as alternatives and the fact that they cannot be included in a single regression. Except for the high correlation among sustainable energies, none of the control variables have a high correlation to possess potential multicollinearity issues.

**Table 3:** Correlation matrix.

	1	2	3	4	5	6	7	8	9
Clean energy (total)	1								
Clean energy (Urban)	0.94	1							
Clean energy (Rural	0.93	0.8	1						
China investment	0.15	0.15	0.12	1					
Gross domestic product	0.34	0.34	0.31	0.68	1				
GDP per capita	0.76	0.75	0.62	0.25	0.28	1			
GDP growth	−0.16	−0.17	−0.13	−0.07	0.04	−0.12	1		
Gross domestic savings	0.34	0.29	0.23	0.3	0.41	0.54	0.09	1	
Domestic credit	0.62	0.57	0.6	0.14	0.3	0.5	−0.12	0	1
Trade openness	0.33	0.34	0.27	−0.06	−0.28	0.47	−0.01	0.21	0.12

Having established the validity of the dataset for regression estimations, we moved to identify the appropriate econometric estimation strategy. To do this, we perform the Hausman test [33] to choose between random or fixed effect modelling. We select between random and fixed because the sample is panel data. The un-tabulated results of the Hausman test lead to the rejection of the null hypothesis that there is no correlation between the unique errors and the repressors in the model. Therefore, the fixed effect is more appropriate than the random effect. We specify our baseline model as follows;

(1) $${\text{Chinese}\;\text{investment}}_{\text{it}}=\alpha_{i}+{\beta_{j}\text{sustainbel}\;\text{energy}}_{\text{ijt}}+X_{\text{it}}^{\prime }\theta +\varepsilon_{\text{ijt}}\;i=1,\ldots ,N\;;\;t=1,\ldots ,T\;;\;j=1,\ldots ,J$$

where $X_{\text{it}}^{\prime }$ is a vector of control variables, respectively, for country I and time t. ${\text{Sustainable}\;\text{energy}}_{\text{ijt}}$ is indicator j of country i at time t and $\varepsilon_{\text{ijt}}$ is the associated error term. We used three measurements of sustainable energy, namely total, urban and rural sustainable energy.

## 3 Results and discussion

### 3.1 Summary statistics

The descriptive statistics of the variables are presented in Table 4. The table includes the mean, standard deviation, and the 25th, 50th (median), and 75th percentiles. The mean of the log form of Chinese investment is 4.385, which is equivalent to 469.3 million USD. This suggests that, over the sample period, China has invested close to half a billion dollars annually in Africa. The standard deviation of Chinese investment is 2.93 (in natural logarithm form), indicating significant variation among the sample. The mean of clean and sustainable energies (total) is 27.81 %, implying that about a quarter of the energy sources in the sample countries over the sample period are generated from clean sources. We observe that the mean is higher in urban areas than in rural areas. We also observe significant variation across the sample countries, as evidenced by the large standard deviation of 33.68 and a low 50th percentile (median) of 10.25.

**Table 4:** Summary statistics.

VARIABLES	(2)	(3)	(4)	(5)	(6)
	mean	sd	p25	p50	p75
China investment (log)	4.385	2.398	2.723	4.709	6.117
Clean fuel and technology (Total)	27.81	33.68	2	10.25	43.30
Clean fuel and technology (rural)	17.96	31.76	0.400	1.450	18.30
Clean fuel and technology (Urban)	39.02	37.02	5	23.75	77.50
Domestic credit	22.39	23.54	9.831	15.37	24.15
Trade openness	71.97	39.85	46.02	62.31	87.25
GDP growth	4.014	4.756	2.247	4.283	6.347
Gross domestic savings	16.27	17.17	6.501	14.56	23.03
GDP per capita (log)	7.215	1.023	6.468	7.066	8.023
Gross domestic (log)	23.14	1.620	22.04	23.17	24.18

### 3.2 Baseline results

The results of the baseline fixed-effect estimations are presented in Table 5. For robustness, we present the results in three columns based on different measures of the dependent variable. Column 1 contains the results for the clean and sustainable energy sources of the whole country. In column 2, the dependent variable captures clean and sustainable energies in urban areas, while column 3 contains the results for clean and sustainable energies in rural areas. Although the correlation matrix in Table 4 shows that these three measures are highly correlated, they capture different access levels to clean and sustainable energies. Hence, presenting separate estimations explains how Chinese investment impacts rural and urban areas. The variable of interest in all three columns is Chinese investment.

**Table 5:** Baseline results.

VARIABLES	(1)	(2)	(3)
	Total	Urban	Rural
China Investment	0.643^a^	0.240^a^	0.715^a^
	(3.252)	(2.914)	(3.893)
Gross domestic product	6.385^a^	11.749^a^	1.282^a^
	(3.568)	(4.950)	(2.771)
GDP per capital	−4.931^b^	−8.022^a^	−0.910
	(-2.527)	(-3.099)	(-0.502)
GDP growth	−0.051	−0.057	−0.038
	(-1.369)	(-1.166)	(-1.103)
Gross domestic savings	−0.115^a^	−0.089^a^	−0.108^a^
	(-5.226)	(-3.029)	(-5.258)
Domestic credit	−0.000	0.093^b^	−0.094^a^
	(-0.003)	(2.396)	(-3.457)
Trade openness	0.069^a^	0.086^a^	0.033^a^
	(5.233)	(4.885)	(2.681)
Constant	−81.669^a^	−174.205^a^	−2.033
	(-2.834)	(-4.557)	(-0.076)
Observations	701	701	701
R-squared	0.317	0.314	0.170
Number of countries	43	43	43

t-statistics in parentheses.

*p < 0.1.

a p < 0.01.

b p < 0.05.

The coefficient of Chinese investment in all three columns is positive and highly significant at the 1 percent level. The result suggests that Chinese investment in Africa increases access to clean fuel and technology. The results support our expectations that the flow of Chinese investment in Africa is associated with an increase in access to clean and sustainable energies. Over the years, China has invested heavily in different energy projects in Africa (China Daily, 2022; Voice of America, 2023). For instance, China has been involved in funding and building large-scale hydropower projects in some African countries, such as the Bui Dam in Ghana. Furthermore, our results are consistent with prior studies (Ofosu & Sarpong, 2022) that indicate Chinese investments often include technology transfer and capacity-building components, such as training local technicians and engineers to operate and maintain sustainable energy infrastructure. Importantly, some Chinese investments in sustainable energy align with African countries' own national development plans and renewable energy targets, fostering a sense of collaboration. Our results are consistent with Zakari and Khan, (2022), who found that Chinese investment boosts energy production in Africa.

Although Chinese investment increases access to clean and sustainable energies, the relationship is more pronounced in rural areas than in urban areas. This is evidenced by the larger coefficient of Chinese investment in column 3. Economically, all other things being equal, a 1-log form increase in Chinese investment will increase access to clean fuel and technology by 1.714 % of total energy consumption in rural areas. The same amount of investment will lead to less than a 1 % (specifically 0.575 %) increase in clean and sustainable energies in urban areas.^2^ This shows that the impact of Chinese investment in energy is more significant in rural areas of Africa. The result is consistent with Lema et al. (2021), who found that the benefit of Chinese investment is locally bounded. This is unsurprising, given that China has been a major player in promoting solar energy in Africa, with initiatives like the Solar Power Plan for Africa. These efforts aim to provide off-grid and decentralised solar solutions to remote and underserved areas (Voice of America, 2023). Chinese financing, often provided through state-owned banks and institutions, has supported the development of sustainable energy projects that might have struggled to secure funding through traditional channels (Alden, 2015; Shen, 2015).

The results for most control variables are consistent with prior studies and standard assumptions (Edziah et al., 2022; Zakari & Khan, 2022). For example, gross domestic product, representing economic size, is positively associated with an increase in clean fuel and technology. Arguably, large economies have the resources to provide clean fuel and technology to a larger population. Similarly, we find that trade openness increases clean and sustainable energies. However, we did not find a significant relationship between GDP growth and clean fuel and technology.

### 3.3 Endogeneity (GMM 2SLS)

Endogeneity arises from various sources, such as year and country effects, reverse causality, omitted variable bias, and prior year effects. Reverse causality is less of a concern because it is unlikely that sustainable energies would attract foreign direct investment from China. Furthermore, the fixed-effect econometric modelling mitigates the effects of year and country covariates on estimation. The baseline models also mitigate omitted variable bias [33]. However, there is still a potential endogeneity issue due to the contemporaneous effect of the dependent variable and the dynamic nature of the relationship between Chinese investment and sustainable energy. To address these concerns, we employ the two-step System Generalized Method of Moments (S-GMM) to check the robustness of the results. The System Generalized Method of Moments uses additional moment conditions and tackles the problem of weak instruments often encountered with other instrumental modelling methods, such as the Generalized Method of Moments and Two-Stage Least Squares [34]. The S-GMM also mitigates the contemporaneous effect of the dependent variable by differencing and using lagged values as instruments for the endogenous variable (Arellano & Bond, 1991; V. Tawiah et al., 2022).

The results of the S-GMM are presented in Table 6. Similar to the baseline results, we present the estimations for the three dependent variables: access to sustainable energy (overall), urban areas, and rural areas. The coefficient of Chinese investment remains positive and significant in all three columns of Table 6, confirming our baseline results that Chinese investment increases access to sustainable energy in Africa. These results imply that our findings are not sensitive to potential endogeneity.

**Table 6:** Endogeneity check – SGMM.

VARIABLES	(1)	(2)	(3)
	Total	Urban	Rural
China Investment	−2.862^a^	−2.542^a^	−3.157^a^
	(-33.959)	(-17.217)	(-42.118)
Gross domestic product	5.047^a^	5.910^a^	7.552^a^
	(13.513)	(10.166)	(54.889)
GDP per capital	17.777^a^	21.213^a^	9.712^a^
	(54.572)	(19.559)	(30.083)
GDP growth	−0.335^a^	−0.406^a^	−0.239^a^
	(-37.004)	(-13.043)	(-42.481)
Gross domestic savings	−0.025^a^	−0.233^a^	−0.085^a^
	(-3.064)	(-14.754)	(-10.236)
Domestic credit	0.436^a^	0.327^a^	0.480^a^
	(36.082)	(18.520)	(49.902)
Trade openness	0.013^a^	0.040^a^	0.020^a^
	(2.616)	(6.536)	(8.136)
Constant	−213.836^a^	−246.102^a^	−223.882^a^
	(-30.250)	(-20.468)	(-53.484)
AR (1)	0.023	0.026	0.004
AR (2)	0.556	0.764	0.912
Hasen	0.896	0.864	0.749
Observations	701	701	701
Number of ID	43	43	43

z-statistics in parentheses.

*p < 0.1.

**p < 0.05.

a p < 0.01.

### 3.4 Moderating effect of natural resources

One of the major criticisms of China's presence in Africa is the rapid exploitation of natural resources. Many argue that China is primarily in Africa to extract these resources [11,35,36]. This is evidenced by the substantial amount of Chinese investment in resource-rich countries. For instance, Algeria and Angola, which are resource-rich, have been receiving significant Chinese investment (China Africa Research Initiative, 2023). Therefore, the presence of natural resources in a country could influence the impact of China's investment there. Hence, our findings on the positive and significant impact of Chinese investment on access to clean and sustainable energies might be biased due to the combination of both resource-rich and non-resource-rich countries in a single analysis. To address this concern and test whether the relationship between Chinese investment and clean energies differs based on natural resource endowment, we run separate regressions for resource-rich and non-resource-rich countries. Consistent with prior studies [37,38], we group the countries according to the World Bank classification.

Similar to the main findings, we present results for the three different measurements of clean fuel and technology. The results are presented in Table 7. The results for resource-rich countries are presented in columns 1 to 3. The coefficient of China's investment is positive and significant in all these columns, implying that China's investment increases access to clean and sustainable energies in resource-rich countries in both rural and urban areas. Columns 4 to 6 present the results for non-resource-rich countries. As in the baseline results, the coefficient of Chinese investment is positive and significant in all these columns. However, the effect is more pronounced in rural areas than in urban areas. Therefore, the results in Table 7 suggest that the impact of China's investment on access to clean and sustainable energies does not differ significantly between resource-rich and non-resource-rich African countries.

**Table 7:** Resource rich and non-resource rich countries.

VARIABLES	Natural resources			Non-natural resources		
	(1)	(2)	(3)	(4)	(5)	(6)
	Total	Urban	Rural	Total	Urban	Rural
China Investment	0.322^b^	1.011^b^	0.252^b^	0.395**	0.542**	0.985^a^
	(2.142)	(2.525)	(2.042)	(2.540)	(2.101)	(4.172)
Gross domestic product	9.561^a^	7.395^b^	9.478^a^	10.746^a^	18.673^a^	5.232^b^
	(3.938)	(2.140)	(4.535)	(4.430)	(5.791)	(2.327)
GDP per capital	−7.779^a^	−6.105^c^	−8.553^a^	−11.120^a^	−15.397^a^	−7.300^a^
	(-3.098)	(-1.709)	(-3.958)	(-3.976)	(-4.142)	(-2.816)
GDP growth	0.024	0.014	0.037	−0.144^a^	−0.135^c^	−0.137^a^
	(0.604)	(0.255)	(1.091)	(-2.621)	(-1.850)	(-2.704)
Gross domestic savings	0.031	0.091^b^	0.011	−0.328^a^	−0.333^a^	−0.307^a^
	(1.200)	(2.510)	(0.505)	(-9.027)	(-6.908)	(-9.115)
Domestic credit	0.156^b^	0.271^a^	−0.084	−0.006	0.083^c^	−0.069^b^
	(2.522)	(3.075)	(-1.566)	(-0.173)	(1.861)	(-2.211)
Trade openness	−0.075^a^	−0.101^a^	−0.023^c^	−0.063^a^	−0.078^a^	−0.035^c^
	(-5.281)	(-5.036)	(-1.852)	(-3.182)	(-2.975)	(-1.919)
Constant	−141.395^a^	−96.114^c^	−144.901^a^	−134.940^a^	−276.789^a^	−43.850
	(-3.543)	(-1.692)	(-4.219)	(-3.561)	(-5.496)	(-1.249)
Observations	287	287	287	414	414	414
R-squared	0.537	0.517	0.181	0.345	0.304	0.336
Number of ID	17	17	17	26	26	26

t-statistics in parentheses.

a p < 0.01.

b p < 0.05.

c p < 0.1.

### 3.5 Regional blocs

To this point, our estimations have been based on the assumption that all African countries are similar and likely to receive the same attention from China. However, China's investment in Africa is not uniform across all countries in the region, as each country has its own unique set of opportunities, challenges, and priorities. For instance, China's demand for minerals has led to investments in West and Southern African mining projects, particularly for resources like iron ore, bauxite, coal, diamonds and gold. China's investment in North Africa is dominated by the oil and gas sector. Investment in East Africa is focused on transportation and green energy. These unique dimensions of Chinese investments across Africa indicate that the consequences could also be different based on the regional location of the country. Therefore, in this section, we run separate regressions on a sub-sample group based on the country's regional location. We sub-sample the countries into four (4) regional groups: North, South, East and West.

The results are presented in Table 8. The dependent variable is total clean fuel and technology. Columns 1, 2, 3 and 4 contain the estimations of North, South, East and West Africa countries. The coefficient of China's investment is positive and significant at 5 % or better, suggesting that the positive influence of China's investment in increasing access to clean fuel and technology is not sensitive to the regional location of the country. However, we observe that the coefficient of East African countries is larger, followed by West Africa and South Africa, with North Africa having the smallest. This is unsurprising, given China's investment in green energy in most of East Africa. For instance, the Garissa photovoltaic power generation project in Kenya is currently the largest photovoltaic power station in East Africa, with an average annual power generation output of 76 million kilowatt-hours. There is also an Aysha wind power project in Ethiopia. China's investment in North Africa is in the energy sector, but it is predominately in the oil and gas sector, which is not considered clean energy. Although the Southern Africa region has some clean energy investment from China, the large ones are still under construction. For instance, a wind farm in Namibia and a floating solar farm on Zimbabwe's massive Kariba Dam are among the new green energy projects Chinese companies are developing in Southern Africa [9].

**Table 8:** Regional bloc.

VARIABLES	(1)	(2)	(3)	(4)
	North	South	East	West
China Investment	0.233**	2.518***	0.358**	0.482*
	(2.466)	(5.257)	(2.233)	(1.929)
Gross domestic product	2.141	−2.390	21.866***	13.154***
	(0.919)	(-0.526)	(6.844)	(6.037)
GDP per capital	−0.101	−2.455	−19.598***	−9.664***
	(-0.041)	(-0.527)	(-5.619)	(-4.032)
GDP growth	−0.009	0.000	0.038	−0.017
	(-0.245)	(0.004)	(0.462)	(-0.422)
Gross domestic savings	−0.056**	−0.099*	−0.667***	0.038*
	(-2.019)	(-1.788)	(-14.362)	(1.657)
Domestic credit	−0.038**	0.024	−0.221***	−0.065
	(-2.576)	(0.532)	(-3.550)	(-1.177)
Trade openness	0.011*	−0.097***	−0.111***	−0.104***
	(1.827)	(-3.202)	(-4.082)	(-6.465)
Constant	37.862	102.909	−333.896***	−207.831***
	(0.950)	(1.361)	(-6.510)	(-6.110)
Observations	72	131	177	321
R-squared	0.804	0.395	0.734	0.403
Number of countries	5	9	10	19

t-statistics in parentheses.

***p < 0.01, **p < 0.05, *p < 0.1.

## 4 Conclusion

Chinese foreign investment in sustainable energy in Africa has been a significant and evolving aspect of the relationship between China and African countries. China has been involved in various initiatives, projects, and partnerships to promote sustainable energy development on the continent. However, the outcomes and impacts of these investments can vary based on specific projects, policies, and local contexts. Therefore, in this paper, we have examined the impact of Chinese investment on clean and sustainable energy in Africa.

Employing robust econometric modelling on a large panel sample of 43 countries over 19 years, we find a positive and significant relationship between Chinese investment and access to sustainable energy in Africa. The results suggest that over the last decades, the inflow of Chinese investment into Africa has contributed significantly to producing clean and sustainable energy. This aligns with China's commitment to delivering its international climate pledges by promoting energy transition from fossil fuels in Africa (Voice of America, 2023). We also extend the literature on the impact of foreign direct investment on renewable energy (Aluko et al., 2023; Lema et al., 2021; Mbiankeu Nguea et al., 2022; Zakari & Khan, 2022). However, we provide new insights by focusing on FDI from a single dominant country, which overcomes the challenges of analysing investments from multiple countries with different objectives.

Our study builds on prior research on energy supplies [39,40] and offers several policy implications. We provide empirical evidence on how Chinese investment influences sustainable energy production and consumption in Africa. By extension, we show how China's presence in Africa contributes to sustainable development and the attainment of the Sustainable Development Goals (SDGs) and Agenda 2030 [41]. report a negative relationship between energy security and poverty reduction. Hence, access to clean and sustainable electricity is crucial for achieving various SDGs, including those related to poverty alleviation, health, education, and economic growth. The findings suggest that Chinese investment has the potential to contribute positively to these goals in emerging economies. Policymakers should prioritise policies and initiatives that align with the SDGs and leverage Chinese investment to advance sustainable development objectives.

The results also inform policymakers about the relevance of Chinese investment in the development of clean and sustainable energy in Africa and other host countries. The positive association between Chinese investment and access to clean and sustainable energy underscores the importance of collaboration and partnership between China and African countries. Policymakers should prioritise fostering mutually beneficial partnerships and collaborations that promote sustainable energy development. This could involve initiatives such as joint research projects, technology-sharing agreements, and public-private partnerships focused on renewable energy initiatives.

Notwithstanding the positive results of Chinese investment, there are potential downsides to China's presence in Africa [42]. Some critics argue that China's investments in Africa, including energy projects, have led to debt-related challenges for recipient countries [43,44]. If countries become heavily indebted to China, it could affect their ability to invest in other critical sectors, including social services like healthcare and education, which could indirectly impact sustainable development and energy access.

There are also concerns about the ownership and control of energy infrastructure developed through Chinese investments [45,46]. In some cases, Chinese companies may have a significant role in managing and operating these projects, potentially affecting the sovereignty and control of the host country over its energy resources [42]. Furthermore, Chinese investments often bring in foreign labour for construction and operation, which might limit local job creation and skill development. However, some projects involve knowledge transfer and training programs for local workers, contributing to capacity building. Therefore, it's crucial for African countries to carefully assess and manage the terms of these investments to ensure long-term sustainability, local benefits, and environmental considerations. Collaborative efforts between China, African governments, and other stakeholders are important to ensure that energy investments contribute positively to sustainable development goals.

As with many empirical studies [25,47], this paper is limited in considering the laws and terms of engagement between China and Africa. Therefore, future studies can examine the role of governance structures, institutional capacity, and policy frameworks in shaping the impact of Chinese investment on energy access in Africa. Such studies could analyse how factors like transparency, accountability, local participation, and regulatory enforcement influence the effectiveness and sustainability of energy projects. Other studies could evaluate the effectiveness of existing policies, regulations, and governance mechanisms in promoting sustainable energy development and maximising the benefits of Chinese investment, identifying policy gaps, regulatory challenges, and opportunities for strengthening the enabling environment for energy access initiatives. Future research can also explore the impact of Chinese investment in other sectors, such as transportation and agriculture.

## Funding information

Princess Nourah bint Abdulrahman University Researchers Supporting Project number (PNURSP2024R391), Princess Nourah bint Abdulrahman University, Riyadh, Saudi Arabia.

The General Project of Jiangsu Social Science Fund “Research on Legal Protection Mechanism of Ecological Compensation in Main Functional Areas” in 2021 (project number 21FXB007). The Major project of philosophy and social science research in Jiangsu universities, “Study on the Control of Ecological Space Use in Nature Reserves in 2021 (Project number: 2021SJZDA146).

## Data availability

Has data associated with your study been deposited into a publicly available repository? **NO**.

Has data associated with your study been deposited into a publicly available repository?

Data will be made available on request.

### Ethical statement

Ethical compliance is not applicable.

## CRediT authorship contribution statement

**Dejun Zhou:** Writing – review & editing, Supervision, Resources, Project administration, Funding acquisition. **Noha Alessa:** Writing – review & editing, Funding acquisition, Conceptualization. **Vincent Konadu Tawiah:** Writing – original draft.

## Declaration of competing interest

The authors declare that they have no known competing financial interests or personal relationships that could have appeared to influence the work reported in this paper.

## Footnotes

1 China Africa Research Initiative (2023). Data: China-Africa Trade. http://www.sais-cari.org/data-china-africa-trade.

2 Consistent with prior studies [48,49], we calculate economic significance as coefficient*standard deviation.
